# Liver Histopathology in Late Protocol Biopsies after Pediatric Liver Transplantation

**DOI:** 10.3390/children8080671

**Published:** 2021-08-01

**Authors:** Małgorzata Markiewicz-Kijewska, Sylwia Szymańska, Michal Pyzlak, Piotr Kaliciński, Joanna Teisseyre, Adam Kowalski, Irena Jankowska, Piotr Czubkowski, Hor Ismail

**Affiliations:** 1 The Department of Pediatric Surgery and Organs Transplantation, The Children’s Memorial Health Institute, 04-730 Warsaw, Poland; p.kalicinski@ipczd.pl (P.K.); j.teisseyre@ipczd.pl (J.T.); a.kowalski@ipczd.pl (A.K.); h.ismail@ipczd.pl (H.I.); 2 The Department of Pathology, The Children’s Memorial Health Institute, 04-730 Warsaw, Poland; 3 The Department of Pathology and Laboratory Diagnostic, Maria-Sklodowska-Curie National Research Institute of Oncology, 02-781 Warsaw, Poland; mpyzlak@patomorfologia.com; 4 The Department of Gastroenterology, Hepatology, Nutrition Disorder and Pediatric, The Children’s Memorial Health Institute, 04-730 Warsaw, Poland; i.jankowska@ipczd.pl (I.J.); p.czubkowski@ipczd.pl (P.C.)

**Keywords:** pediatric liver transplantation, liver biopsy, histopathology, long term follow-up

## Abstract

Liver transplantation has become a routine treatment for children with end stage liver failure. Recently, the long term survival of pediatric patients after liver transplantation has improved, with a life expectancy much longer than that of adult recipients, but also with longer exposition of the graft to various injuries, including immunological, inflammatory and others. Biochemical tests, although important, do not always reflect graft injury. The aim of our study was to analyze the histopathology of the graft in late protocol biopsies and correlate it with the clinical and biochemical status of these patients. We analyzed 61 protocol liver biopsies taken from 61 patients. Biopsies were taken 9.03–17.09 years (mean 12.68, median 11.74 years) after transplantation. Liver specimens were examined particularly for the presence and stage of liver fibrosis, inflammation, steatosis, and acute or chronic cellular and humoral rejection. We did not find any abnormalities in 26 (42.6%) liver specimens. None of the patients had signs of cellular or antibody mediated rejection or chronic rejection. In 23 liver biopsies (37.7%), we found non-specific lymphoid infiltrates. Another problem was fibrosis (equal to or more than three on the Ishak scale)—we found it in 17 patients, including seven liver specimens (11.5%) with severe fibrosis (Ishak 5–6). Conclusions: Various pathomorphological abnormalities were found in more than half of patients with a median 11.74 years post-transplant follow-up. Most of them presented normal laboratory liver tests at the same time, suggesting a slow subclinical process leading to pathomorphological abnormalities. No single factor for the development of these abnormalities was found, but our study supports the need for protocol liver biopsies even in patients with normal/almost normal biochemical liver tests.

## 1. Introduction

The transplantation of the liver is currently one of the most effective ways to treat the irreversible failure of this organ. The quality of life of patients after transplantation is good and the survival time in the first year is 85% and more for patients with low-risk (up to 95%) [1]. It is related not only to more and more effective immunosuppressive agents and regular follow-up visits but also to innovative surgical methods including the usage of reduced, split, and living related liver grafts. Patients who were transplanted in childhood are alive and well up to 10–15 years after transplantation, which is crucial especially in the youngest cases [1].

Nevertheless, in most cases we are not able to assess liver histology and function after children grow up and are transferred to the care of adult specialists. The gold standard of the assessment of the histopathological status of the liver is a needle core biopsy. In the literature, there are increasing data documenting histological abnormalities in late biopsies of pediatric liver transplantation (LT) in the context of (near) normal liver biochemistry [2,3,4,5,6,7]. These changes include chronic hepatitis, fibrosis and steatosis, and they are often progressive and may even possibly represent a form of chronic rejection [8].

The longer the time after transplantation, the more likely it is that histopathological changes to different extents will be found in biopsy. Therefore, several authors propose to perform protocol biopsies performed at various follow ups, mostly 1, 5, 10, 15 years after transplantation and at the age of 18 just before transition to adult care [9]. Analyses of protocol liver biopsies performed in predominantly adult liver transplant recipients have revealed abnormal histology in 36% to 88% of patients. Data concerning children are limited, mainly due to the concern about submitting children to invasive procedures, particularly if the liver function assessed by biochemical tests seems to be normal. It was proved, however, that graft histology is associated with both graft and patient survival [10]. According to a study of a cohort of pediatric liver transplant recipients who underwent protocol liver biopsies, chronic hepatitis was the most common histological abnormality as well as fibrosis of various severities.

Time from LT was a factor that increased the risk of fibrosis. In another study, 158 asymptomatic children who underwent protocol biopsies at 1, 5 and 10 years after transplantation were analyzed for histopathological changes. The obtained results were compatible with the observations of Evans et al. [11]. One of the most common abnormalities was fibrosis, which was present in 22%, 43% and 64% of biopsies at 1, 5 and 10 years, respectively (*p* < 0.0001). More importantly, the probability of significant fibrosis increased with time after the procedure—52%, 81% and 91% at 1, 5 and 10 years, respectively (*p* < 0.0001)—and, with post LT follow up longer than 10 years, fibrosis reached the degree of cirrhosis in 15% of cases. Therefore, it seems very important to perform biopsies regularly even when the results of laboratory tests are normal, especially in patients transplanted in infancy or early childhood with the longest predicted expectancy of living with the liver graft. This is particularly interesting in view of the widely accepted tendencies to reduce immunosuppression to the lowest possible levels and completely avoid corticosteroids in children.

The aim of our study was to assess the histopathology of the liver grafts in biopsies taken after long term follow-up with pediatric LT recipients. It is mandatory to understand the mechanisms that cause injury to the organ and determine the phenotypes predisposed to a worse prognosis to optimize the management of pediatric patients after liver transplantation. We also tried to analyze the possible effects of the microscopic changes on graft survival.

## 2. Materials and Methods

Between 1990 and 2021, we performed 848 pediatric liver transplantations in the Children’s Memorial Health Institute. A protocol liver biopsy program after liver transplantation started in 2016. Patients enrolled in this study had liver transplantation between June 2000 and January 2010.

In this study, we analyzed 61 late protocol liver graft biopsies taken from 61 pediatric liver transplant recipients. Biopsies were taken after a follow-up of 9.03–17.09 years (mean 12.68, median 11.74 years) after transplantation. The most common indication for transplantation was biliary atresia (41 patients, 67.2%) then other cholestatic liver diseases for 7 patients, cryptogenic cirrhosis in 3 patients, acute liver failure (ALF) in 2 patients, graft failure in 2 patients and other reasons for 6 patients. In 38 patients, transplantation was performed electively, in 15 patients with acute decompensation of chronic liver disease, in 5 patients urgently, and in 3 children due to oncological reasons (primary malignant liver tumors). Patients were transplanted at ages between 0.12 and 6.87 years (mean 2.02 years, median 1.3 years). Forty seven children (77%) received grafts from living related donors and 14 patients from deceased donors. All patients presented with normal or near normal liver function tests at the time of graft biopsy (ASP and ALT no more than twice the normal range). For the study, we divided patients into 2 groups depending on the findings in the liver biopsy specimens. Group 1 included children with normal or almost normal (borderline) liver biopsies and group 2 consisted of patients with different abnormalities in their liver biopsies.

### 2.1. Histopathology

All tissue samples were fixed in 4% formalin and embedded in paraffin. The paraffin 4µm sections were routinely stained with hematoxylin and eosin (H&E). Following immunohistochemical staining, in each specimen the following were performed: C4d (Biomedica group, dilution 1:40) as a marker of antibody-mediated rejection (AMR) as well as histochemical staining with azan to assess the extent of fibrosis. Additional histopathological changes were re-evaluated, including features of cellular and/or humoral rejection; severity of fibrosis (Ishak scale) [12,13]; presence of cholestasis and steatosis. All liver biopsies (all stainings) taken from liver transplant patients, who were enrolled in the study, were evaluated by the same pathologists. The pathologists assessing liver biopsies knew when the patient was transplanted and that it was a protocolar liver biopsy.

### 2.2. Statistical Analysis

Data were analyzed using the Statistica13.3 software, StatSoft (Polska, 30-110 Kraków, Poland). The analysis involved the assessment of baseline demographics and clinical data using median ranges and distributions for categorical variables. The Mann–Whitney U test was used to assess unpaired associations between continuous variables.

### 2.3. Ethical Approvement

The study was conducted in accordance with the principles of the Declaration of Helsinki. The study was approved by the Ethics Committee at the Children’s Memorial Health Institute (approval number: 19/KBE/2017). Informed consent was signed in accordance with the above guidelines. The research was conducted within the framework of study no. 248/17.

## 3. Results

We assessed 26 liver biopsies in group 1 and 35 liver biopsies in group 2. Group 1 included 26 children with normal or almost normal liver biopsies—biopsies that were borderline (minimal fibrosis: Ishak 0–2, no or minimal inflammatory infiltrates, cholestasis or steatosis). Group 2 consisted of 35 pts with various abnormalities found in the graft biopsies. Details of evaluated groups are presented in Table 1.

There were no signs of acute or chronic cellular rejection, as well as antibody mediated rejection (C4d negative in all biopsies, no portal microvascular endothelial cell enlargement), in any biopsy. De novo autoimmune hepatitis was not diagnosed in any of these patients.

**Figure 1:**
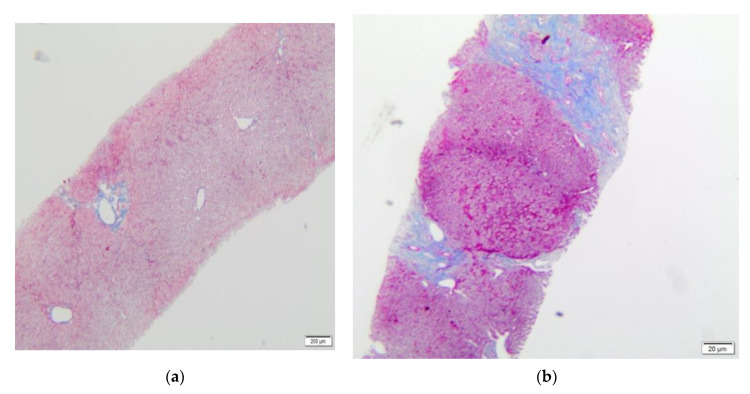
Liver biopsy—(**a**) histochemical staining AZAN (blue)—Ishak 1. (**b**) Liver biopsy—histochemical staining AZAN (blue)—Ishak 4–5.

In group 1 there was no or only minimal pericentral veins fibrosis (Ishak 0–2, mean 1, median 1), (Figure 1a). Ishak 1 fibrosis was found in nine biopsies (34.6%) and Ishak 2 in six biopsies (23%). Fibrosis was not found (Ishak 0) in ten biopsies (42.4%). Minimal cholestasis in hepatocytes (single hepatocytes with bilirubin deposits) was present in two biopsies. Minimal lymphocytic infiltration was present in two patients. We did not find steatosis in any biopsy.

At the time of liver biopsy, 20 patients remained on monotherapy with: tacrolimus (16 patients), sirolimus, (three patients) and cyclosporin (one patient). Only six patients were on double drug immunosuppression (tacrolimus+MMF in three patients; cyclosporine + MMF in one patient; tacrolimus + CS in one patient; and MMF + CS in one patient). As a result of the liver biopsy, we did not change immunosuppression treatment in any patient from group 1.

**Figure 2:**
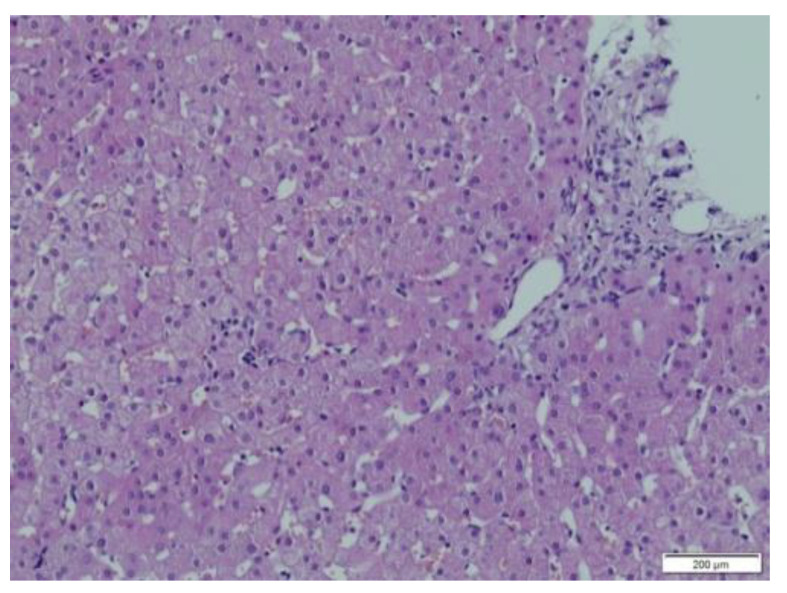
Liver biopsy specimen—H&E—minimal lymphocytic infiltration.

**Figure 3:**
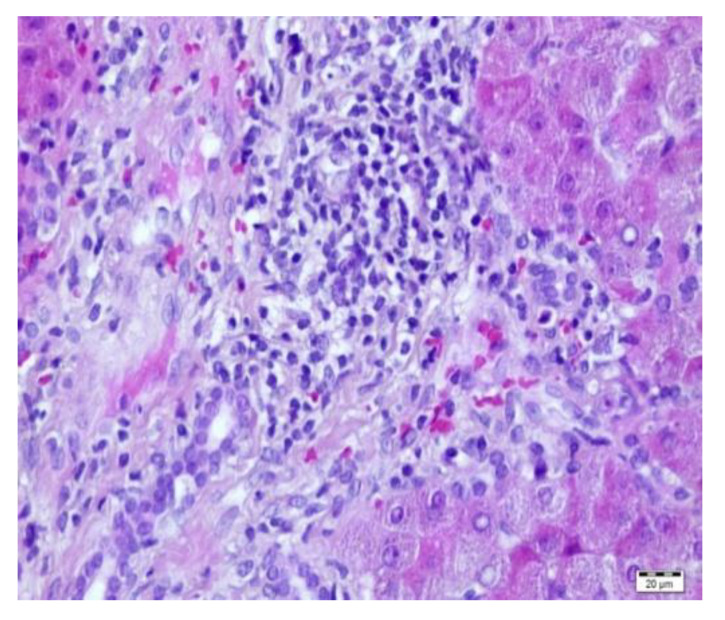
Liver biopsy—H&E—nonspecific inflammation.

In patients from group 2, the range of fibrosis was assessed between 0–6 by Ishak score (mean 3, median 3) (Figure 1a,b). Ishak 1 fibrosis was found in four biopsies (11.4%) and Ishak 2 was present in eight biopsies (22,8%); however, in all of these patients with low Ishak scores, other significant abnormalities were also found. Cholestasis was found in 11 patients: minimal in eight patients; mild in two patients; and severe in one patient. Steatosis was present in six biopsies: minimal (5%–10%) in four patients; mild (25%) in one patient; and severe (60%) in one patient. Non-characteristic inflammatory lymphocytic infiltrations (not fulfilling ACR criteria) were seen in 21 biopsies: minimal in 12 patients; moderate in seven patients; and severe in two patients (Figure 2 and Figure 3). Ishak scores between both groups were significantly different; *p =* 0.00009 (Mann-Whitney test). Table 2 shows the comparison of histopathological changes in liver biopsies in both groups.

At the time of liver biopsy, 25 patients from group 2 remained on monotherapy, with 20 patients on tacrolimus, three patients on sirolimus and two patients on low dose corticosteroids (after discontinuation of immunosuppression due to previously diagnosed PTLD or lymphoma). Only six patients were on double immunosuppression (tacrolimus + MMF in two patients; tacrolimus + CS in two patients; tacrolimus + AZA in one patient; and SRL + CS in one patient). Four patients remained on triple immunosuppression (tacrolimus + AZA + Cs—three patients, tacrolimus + MMF + CS—one patient).

Immunosuppressive treatment was modified in 20 patients from group 2. In six patients, another immunosuppressant (usually corticosteroids, and in one patient sirolimus), was added. In ten patients, the dosage of immunosuppressive drugs was increased and in another two patients, CNI (tacrolimus) was changed to sirolimus. In two patients, tacrolimus was switched to a long acting formulation.

Comparing immunosuppressive therapy between both groups, we did not find differences, which could explain the development of histopathological abnormalities, particularly fibrosis and other changes in patients from group 2. There were no differences in immediate post-transplant immunosuppression, or in the duration of treatment with corticosteroids between transplantation and liver biopsy. Target levels of tacrolimus after 1 month, 1 year, 5 years and 10 years was also similar. Immunosuppression details are presented in Table 3.

There was no difference in the incidence of acute cellular rejection between both groups. Episodes of ACR occurred in 11 pts (25 episodes; 42.3% pts) from group 1 and in 17 pts (30 episodes; 48.6% pts) from group 2. In the statistical analysis of ACR episodes at different times after transplantation, there were no statistical differences between both groups (Table 4).

There were no differences in biochemical parameters (bilirubin concentration, ASPAT, ALAT, GGTP) between both groups. Comparing biochemical parameters at the biopsy with those taken at the last follow up, there is a slightly increased maximum ALAT activity in both groups but without change in median value. (Table 5)

## 4. Discussion

Performing protocol biopsy in patients with normal results of laboratory tests who do not present any symptoms is still a controversial issue. On the other hand, many authors [4,6,7,9,11,14,15,16,17,18] have described histopathological changes in children with a good graft function. Until now, it is not entirely clear what the reason is for these clinically silent histopathological abnormalities and what significance this type of changes has for the prognosis of long term graft function. Our study was based on the analysis of results of protocol biopsies taken after long post-transplant follow-ups, ranging from 9 to 17 years (median 11.7 years).

The most common histopathological change in our group was non-specific lymphoid infiltrates. Relatively often, minimal to moderate fibrosis was also seen. This observation was consistent with the report of Kelly et al. [19]. Severe fibrosis (Ishak 5–6) was rarely observed, which, in the context of long-term follow-up, is a positive observation. However, in contrast to the research of Sebagh et al. and Evans et al., who presented a low number of biopsies with normal histology, among our patients many more did not show any irregularities in the liver biopsy (42% in our study vs. 20% and 7.4% in other papers) despite longer post-transplant follow up at the time of biopsy [11,20]. On the basis of these observations, it can therefore be deduced that, after a critical period, children who have effective immunosuppression maintain not only the optimal results of laboratory tests but also do not develop changes at the histological level. We referred histological results to clinical and laboratory data to correlate them, trying to find parameters which could potentially reflect pathomorphologic changes, but neither the incidence of previous episodes of acute cellular rejections (ACR) nor a diagram of administered immunosuppression differentiated patients from groups 1 and 2.

Ekong et al. proved that advanced fibrosis was associated with time after transplantation (>6 years) and inflammation, which would indicate that the ongoing damage of an allograft favors the development of cirrhosis of the liver graft. In our study, despite a median of 11.7 years of follow-up at biopsy, significant fibrosis (5 according to Ishak) was observed in only six out of 61 (9.8%) examined biopsies, which does not confirm the conclusions of the authors. Ekong also described the correlation between the episodes of rejection and histopathological changes. In our group, we have not confirmed a relationship between ACR and abnormalities in biopsy. None of our patients showed changes suggesting chronic rejection. According to Ekong et al., chronic changes imply incorrect laboratory tests. Our patients, however, have mostly normal liver function tests. [9].

Another important problem is minimal pericentral fibrosis in part of the liver biopsies some years after transplantation (normal/borderline biopsies). In our study, we found it in nine patients from group 1 and in four patients from group 2 (21.3% of all patients). Fouquet et al. described centrilobular fibrosis in 22% patients 10 years after transplantation in protocol biopsies (patients were asymptomatic with normal liver function, and maintained immunosuppression), which is similar to our observation [17]. Egawa et al. suggest that such minimal changes can be related to too low immunosuppression or inadequate immunosuppression under CNI withdrawal. [21]

Over the years, some authors have emphasized the importance of AMR and DSA and their influence on liver fibrosis. It is interesting that some authors observed high levels of DSA (up to 22%) even in patients without episodes of ACR. In some situations, C4d deposits were found in liver biopsy specimens of patients without detectable DSA. It is possible that C4d deposits can be secondary to autoimmune reactions in the liver. They were found in liver biopsies taken from patients with autoimmune hepatitis. It cannot be ruled out that all these processes may contribute to the formation of liver fibrosis [22,23,24].

It is not clear from our study, or from the literature, how we should react to the histopathological abnormalities found in children with normal liver function and long follow-up, particularly in adolescents and those transitioning to adult care. This time itself is difficult for the patients and is often followed by some deterioration in graft function, due to non-compliance in many cases. After excluding other causes of fibrosis, inflammation, cholestasis and steatosis (viral infection, toxic, dietary, de novo AIH etc.), subclinical immunological processes should be taken as most likely. Therefore, most of our interventions in group 2 were based on the introduction of low dose CS to previous immunosuppression (particularly in patients with advanced fibrosis) or the increase of basic immunosuppressive drug dosage (particularly in patients with advanced inflammation) or both in the case of multiple abnormalities in biopsy. Some authors suggested repeating liver biopsy after a few years and in all situations with the deterioration liver function [17,21]. Martinelli et al., in his study of patients 20 years after LT, found the deterioration of liver tests in 30% of patients to be the main problem, and changes in liver biopsies were mainly in the form of fibrosis. [25] We plan to repeat biopsies within 2 years in all patients after interventions to check whether there is any improvement in histopathologic examination. Preliminary data on a few patients show a decrease in fibrosis score, but more data are necessary to confirm this observation.

## 5. Conclusions

In summary, we have shown in our study that, in more than half of the protocol biopsies taken 10 and more years after transplantation in pediatric patients, various histopathological abnormalities can be found. It is disturbing that some of these changes (e.g., severe fibrosis), which could lead to the subsequent loss of the graft, were found in patients with normal liver function as assessed by biochemical tests. Therefore, it seems to be justified to perform biopsies even many years after the procedure and in patients with normal liver laboratory tests, particularly in children before their transition to adult care. As the cause of these abnormalities, and what they mean for long-term graft survival, is still not known, and is not related to typical post-transplant complications, our study indicates that this process is multi-factorial and requires further longitudinal studies with analysis of the efficacy of certain therapeutic interventions.

## Figures and Tables

**Table 1:** Characteristics of evaluated groups.

Parameter	Group 1	Group 2	*p* Value
Number of pts and evaluated biopsies	26 (42.6%)	35 (57.4%)	
Number of pts after LrdLtx	22 (84.6%)	25 (71.4%)	NS (*p =* 0.385)
Age at LT (range, mean and median)	0.12–6.13 years mean 1.6 median 1.15	0.2–6.87 years mean 2.15 median 1.34	NS (*p =* 0.290)
Body mass at LT (range, mean and median)	4–28 kg mean 10.26 median 10	5–22.5 kg mean 11.05 median 9.55	NS (*p =* 0.502)
MELD/PELD	−9–37 mean 16.31 median 16	−3–53 mean 14.22 median 15	NS (*p =* 0.41)
Time from LT to liver biopsy	9.09–17.01 years mean 12.97 median 12.04	9.03–17.09 years mean 12.47 median 11.47	NS (*p =* 0.307)
Post-transplant follow up (range, mean and median)	11.16–20.69 years mean 15.96 median 15.85	11.79–20.39 years mean 15.28 median 15	NS (*p =* 0.347)

**Table 2:** The extent of histological changes in 61 liver biopsies of patients enrolled in the study.

Ishak Score	Group 1—Number of Patients	Group 2—Number of Patients
0	11 (42.3%)	6 (17.2%)
1	9 (34.6%)	4 (11.4%)
2	6 (23.1%)	8 (22.8%)
3	0	6 (17.2%)
4	0	4 (11.4%)
5	0	4 (11.4%)
6	0	3 (8.6%)
Cholestasis		
minimal	2 (7.7%)	8 (22.8%)
moderate	0	2 (5.7%)
severe	0	1 (2.8%)
Steatosis		
minimal (5–10%)	0	4 (11.4%)
mild (25%)	0	1 (2.8%)
severe (60%)	0	1 (2.8%)
Inflammation		
minimal	2 (7.7%)	12 (34.4%)
moderate	0	7 (20%)
severe	0	2 (5.7%)

**Table 3:** Immunosuppression details.

Parameter	Group 1–26 pts	Group 2–35 pts	*p* Value
Number of IS drugs immediately after LT	1 drug–1 pt 2 drugs–23 pts 3 drugs–3 pts	1 drug–0 pt 2 drugs–34 pts 3 drugs–1 pt	
Induction with monoclonal antibodies	4 pts	7 pts	NS (*p =* 0.653)
Corticosteroids (overall treatment time)	0.07–14.34 years mean 3.54 years median 2.98 years	0.1–9.62 years mean 3.2 years median 2.21 years	NS (*p =* 0.282)
Tacrolimus concentration 1 month after LT	4.9–14.3 ng/mL mean 9.57 ng/mL median 9.6 ng/mL	4.1–19.13 ng/mL mean 8.8 ng/mL median 8.35 ng/mL	NS (*p =* 0.105)
Tacrolimus concentration 1 year after LT	3.5–10.1 ng/mL mean 6.58 ng/mL median 6.7 ng/mL	2.4–13.7 ng/mL mean 6.49 ng/mL median 6.6 ng/mL	NS (*p =* 0.714)
Tacrolimus concentration 5 years after LT	1.1–6.5 ng/mL mean 3.57 ng/mL median 3 ng/mL	1.1–10 ng/mL mean 3.8 ng/mL median 3.4 ng/mL	NS (*p =* 0.79)
Tacrolimus concentration 10 years after LT	1.8–6.3 ng/mL mean 3.68 ng/mL median 3.4 ng/mL	1.1–8 mg/mL mean 3.73 ng/mL median 3.6 ng/mL	NS (*p =* 0.254)

**Table 4:** Incidence of acute cellular rejection.

Parameter	Group 1	Group 2	*p* Value
Number of pts	11	17	NS (*p =* 0.822)
Number ACR episodes	25	30	NS (*p =* 0.915)
Biopsy proven ACR	16	21	NS (*p =* 0.593)
ACR 0–3 months	12	21	NS (*p =* 0.738)
ACR 3–12 months	6	3	NS (*p =* 0.132)
ACR >1 year	7	6	NS (*p =* 0.973)

**Table 5:** Liver biochemical parameters.

Parameter	Group 1	Group 2	*p* Value
Liver biochemical tests at time of liver biopsy (range, mean and median)			
Total bilirubine concentration	0.18–2.69 mg/dL mean 1, median 1	0.21–2.86 mg/dL mean 1, median 1	NS (*p =* 0.053)
ASPAT	14–64 IU/L mean 27, median 26	16–73 IU/L mean 30, median 28	NS (*p =* 0.461)
ALAT	9–29 IU/L mean 18, median 18	11–210 IU/L mean 32, median 20	NS (*p =* 0.151)
GGTP	8–51 IU/L mean 19, median 17	8–623 IU/L mean 65, median 19	NS (*p =* 0.220)
Liver biochemical tests at last follow-up (range, mean and median)			
Total bilirubine concentration	0.2–1.78 mg/dL mean 0.74, median 0.76	0.16–1.56 mg/dL mean 0.65, median 0.59	NS (*p =* 0.385)
ASPAT	12–76 IU/L mean 28.08, median 26.5	15–153 IU/L mean 32.34, median 27	NS (*p =* 0.53)
ALAT	8–57 IU/L mean 22.35, median 18	11–210 IU/L mean 32.43, median 21	NS (*p =* 0.214)
GGTP	8–45 IU/L mean 18.58, median 16	7–623 IU/L mean 61.51, median 18	NS (*p =* 0.31)

## Data Availability

Most relevant data are within the paper. Most the data were taken from patients’ medical records. Since the data collected in the medical records are sensitive and thus, protected by law [the Act on Personal Data Protection and the Medical Records Act], access to these data is limited to healthcare professionals employed in CMHI. All patients’ medical records and other vital information used in the creation of the database are available directly at the Children’s Memorial Health Institute, Warsaw, Poland after contact with the Director of Scientific Affairs, MD PhD Piotr Socha at p.socha@ipczd.pl

## Abbreviations

AB0Ilt	AB0 incompatible liver transplantation
ACR	acute cellular rejection
ALF	acute liver failure
AMR	antibody mediated rejection
AZA	azathioprin
CNI	calcineurin inhibitor
CS	corticosteroids
H&E	hematoxylin and eosin
LT	liver transplantation
MMF	mycophenolate mofetil
